# Silver trauma: is whole body CT warranted in low impact trauma

**DOI:** 10.1093/bjr/tqaf070

**Published:** 2025-03-28

**Authors:** Mark Thomas Macmillan, Rachael Kirkbride, Hui Yen Teh, James Bott, Charlotte Algeo, Christopher Hay, Gregor J A Stenhouse

**Affiliations:** Centre for Cardiovascular Science, University of Edinburgh, Edinburgh, EH16 4SA, United Kingdom; Department of Interventional Radiology, Royal Infirmary of Edinburgh, Edinburgh, EH16 4SA, United Kingdom; Department of Clinical Radiology, Royal Infirmary of Edinburgh, Edinburgh, EH16 4SA, United Kingdom; Department of Clinical Radiology, Royal Infirmary of Edinburgh, Edinburgh, EH16 4SA, United Kingdom; Department of Clinical Radiology, Royal Infirmary of Edinburgh, Edinburgh, EH16 4SA, United Kingdom; Department of Clinical Radiology, Royal Infirmary of Edinburgh, Edinburgh, EH16 4SA, United Kingdom; Department of Interventional Radiology, Royal Infirmary of Edinburgh, Edinburgh, EH16 4SA, United Kingdom; Department of Clinical Radiology, Royal Infirmary of Edinburgh, Edinburgh, EH16 4SA, United Kingdom

**Keywords:** trauma, elderly, whole body CT

## Abstract

**Objectives:**

Trauma in the elderly is associated with high mortality. Elderly people who have suffered low impact trauma, such as fall from standing (FFS) are believed still to be at high risk of injury. Whole body trauma CT (WBCT) is increasingly used to image such people, to prevent missing injuries which are not detected clinically. This study aims to assess the utility of WBCT in assessing elderly people who have suffered FFS.

**Methods:**

Over a 2-year period in a single health board, data were collected retrospectively for all patients that underwent WBCT. Data were collected on the mechanism, pattern of injury, and outcomes including 30-day mortality using clinical records. Comparison was made between pre-CT clinical suspicion and injury found on WBCT to identify discrepancies.

**Results:**

In total, 460 patients were included in this study. Compared with FFS, fall from more than standing was associated with higher adjusted odds of having an injury out with zone of clinical suspicion (Adjusted Odds Ratio (AOR) 2.80, 95% CI 1.23-7.28; *P* = .021). There was no significant difference in 30-day mortality between patients who had an injury on WBCT and those without.

**Conclusions:**

FFS is associated with a reduced risk of injury out with areas of clinical concern when compared with fall from greater than standing. As such, a targeted approach to CT scanning in these patients could be considered.

**Advances in knowledge:**

This study challenges the current prevailing dogma for the requirement of WBCT in elderly people who suffer FFS, providing evidence that such people have a low risk of injuries out with areas of clinical suspicion.

## Introduction

Trauma in the elderly is becoming more common,^1^^,^^2^ the management of these patients can be challenging.^3^^,^^4^ Low energy trauma such as falling from standing or ground level is common in elderly patients and has been recognized to be associated with a high risk of injury and mortality.^5^^,^^6^ Given this, whole body CT (WBCT) from the head to pelvis are increasingly being performed rather than targeted scanning of the areas of concern, even in low energy trauma.^7–9^ The assumption underpinning this is that clinically occult injuries will be detected, resulting in improved outcomes. Despite this, neither the World Society of Emergency Surgery (WSES) nor the UK National Institute for Health Care Excellence (NICE) make specific comments on the use of WBCT in elderly patients.^10^^,^^11^

This study aims to assess the use of WBCT in elderly patients who have suffered low impact trauma from a fall from standing (FFS). We hypothesize that patients who have had an FFS have a lower risk of injury out with the area of clinical concern and therefore could safely undergo a more targeted CT approach.

Additionally, we attempt to determine which factors are associated with mortality and length of hospital stay in elderly patients who undergo WBCT for trauma.

## Materials and methods

This was a retrospective study assessing all patients who underwent WBCT within a single healthcare trust spanning 2 hospitals (including a level 1 trauma centre) between January 2021 and January 2023. Data were collected using electronic records and the radiological archive system.

**Figure 1.**
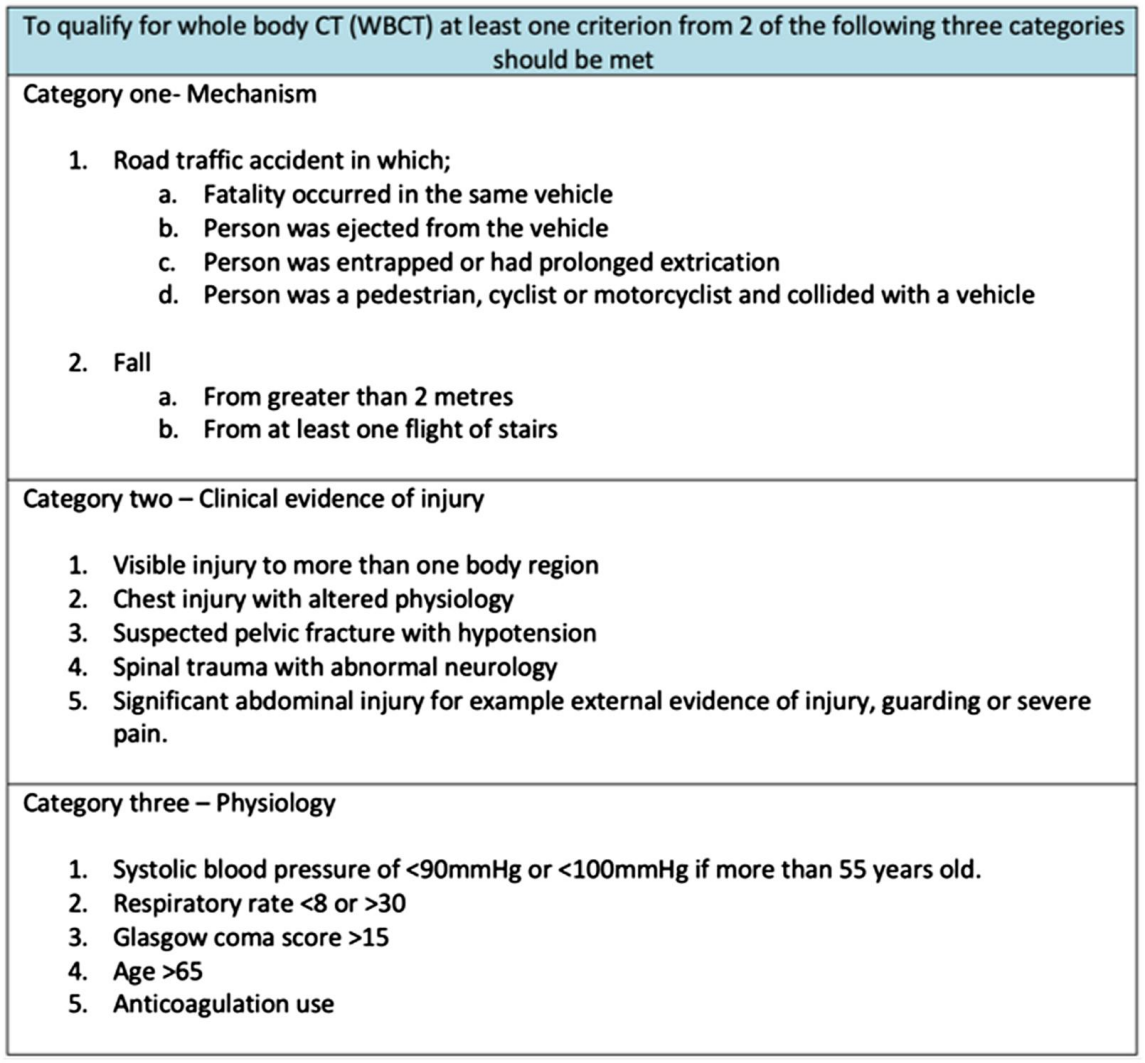
Trauma CT guidance in the study’s health board.

Patients over 70 years old presenting to Accident and Emergency following trauma were included. Patients were excluded who did not have CT of head and neck (non-contrast), and chest, abdomen, and pelvis (split bolus contrast)—our definition of WBCT. Within the study’s health care trust, the guidance for WBCT includes 3 categories namely; mechanism, clinical evidence of injury, and physiological parameters (Figure 1). Each category has a number of criterion, to qualify for WBCT at least one criterion from 2 of the 3 categories must be met. Age of greater than 65 is one of the criterion in the “physiology category.” As such only one criterion from the other 2 categories detailed in Figure 1 was required for patients in this study to qualify for WBCT.

Injuries were categorized into; falls from standing, falls greater than standing, unwitnessed falls with unclear mechanism, road traffic accidents (RTAs), and other mechanisms. The categorization of injury mechanism and suspected injury site(s) was based on the radiological request form and concurrent clinical notes detailing the primary survey prior to performing CT. The suspected injury sites were categorized into head, neck, chest, abdomen, or pelvis to represent the individual segments or “compartments” of a WBCT. This was determined by which CT compartment would most effectively capture the injury. Identified injury sites from the CT were then categorized into head, neck, chest, abdomen, or pelvis. Injuries were those which were included in the conclusion of the radiological WBCT report and indicated as being likely secondary to trauma. This was performed by 1 of 5 radiology trainees (M.T.M., R.K., C.A., J.B., or H.Y.T.).

This categorization allowed us to correlate areas of suspected injury with identified injury on WBCT and identify mismatches.

Injuries out with those detected on WBCT were not recorded. Management strategies pertain only to the injuries identified on WBCT.

Revised trauma score (RTS), which aims to quantify severity of trauma, was calculated using the admission Glasgow coma score (GCS), blood pressure, and respiratory rate.^12^ The score range is 0-12, with 12 being the best and 0 being the worst. This was primarily taken from the doctor’s clinical notes at admission, or if not available, from the ambulance or nursing admission records.

Mortality data were collecting using the electronic patient records. Decision regarding whether mortality had resulted directly from trauma was made by one of the researchers (M.T.M.) based on if reference was made to trauma in the death certificate.

Statistical analysis was performed using R software. Comparisons between categorical variables were made compared using chi-squared testing. Odds ratios were used to compare dependent variables with outcome variables. Adjustments were made for age, sex, cognitive impairment, and anticoagulation use for the adjusted odds model presented in Table 2.

**Table 1.** Baseline characteristics.

		FFS	Fall >than standing	Unwitnessed fall	RTA	Other	*P*-value
		128	227	46	51	8	
Age (*n* = 460)	Median (IQR)	85.5 (79.0 to 90.0)	81.0 (76.0 to 86.0)	83.0 (78.5 to 89.0)	77.0 (74.0 to 81.0)	74.0 (70.8 to 76.0)	<.001
Sex (*n* = 460)	Female	72 (56.2)	100 (44.1)	26 (56.5)	23 (45.1)	2 (25.0)	.089
	Male	56 (43.8)	127 (55.9)	20 (43.5)	28 (54.9)	6 (75.0)	
Cognitive impairment (*n* = 460)	No	107 (83.6)	186 (81.9)	35 (76.1)	49 (96.1)	8 (100.0)	.045
	Yes	21 (16.4)	41 (18.1)	11 (23.9)	2 (3.9)	0 (0.0)	
CT timing (days from injury) (*n* = 441)	<2 days	116 (94.3)	218 (97.3)	33 (91.7)	49 (98.0)	8 (100.0)	.321
	≥2 days	7 (5.7)	6 (2.7)	3 (8.3)	1 (2.0)	0 (0.0)	
Anticoagulation (*n* = 460)	No	61 (47.7)	132 (58.1)	21 (45.7)	38 (74.5)	5 (62.5)	.027
	Antiplatelets	28 (21.9)	49 (21.6)	9 (19.6)	4 (7.8)	2 (25.0)	
	Yes	39 (30.5)	46 (20.3)	16 (34.8)	9 (17.6)	1 (12.5)	
RTS (*n* = 442)	12	102 (85.0)	195 (89.9)	32 (69.6)	46 (90.2)	8 (100.0)	.022
	11	13 (10.8)	13 (6.0)	8 (17.4)	1 (2.0)	0 (0.0)	
	10	1 (0.8)	7 (3.2)	5 (10.9)	3 (5.9)	0 (0.0)	
	9	2 (1.7)	0 (0.0)	1 (2.2)	0 (0.0)	0 (0.0)	
	8	2 (1.7)	2 (0.9)	0 (0.0)	1 (2.0)	0 (0.0)	
Head clinical suspicion (*n* = 460)	No	58 (45.3)	88 (38.8)	12 (26.1)	29 (56.9)	4 (50.0)	.025
	Yes	70 (54.7)	139 (61.2)	34 (73.9)	22 (43.1)	4 (50.0)	
Neck clinical suspicion (*n* = 460)	No	65 (50.8)	133 (58.6)	29 (63.0)	29 (56.9)	4 (50.0)	.55
	Yes	63 (49.2)	94 (41.4)	17 (37.0)	22 (43.1)	4 (50.0)	
Chest clinical suspicion (*n* = 460)	No	25 (19.5)	69 (30.4)	11 (23.9)	10 (19.6)	2 (25.0)	.18
	Yes	103 (80.5)	158 (69.6)	35 (76.1)	41 (80.4)	6 (75.0)	
Abdomen clinical suspicion (*n* = 460)	No	63 (49.2)	110 (48.5)	17 (37.0)	25 (49.0)	2 (25.0)	.414
	Yes	65 (50.8)	117 (51.5)	29 (63.0)	26 (51.0)	6 (75.0)	
Pelvic clinical suspicion (*n* = 460)	No	73 (57.0)	138 (60.8)	28 (60.9)	33 (64.7)	5 (62.5)	.905
	Yes	55 (43.0)	89 (39.2)	18 (39.1)	18 (35.3)	3 (37.5)	
Number of compartments with symptoms (*n* = 460)	0	16 (12.5)	4 (1.8)	0 (0.0)	1 (2.0)	0 (0.0)	.799
	1	1 (0.8)	27 (11.9)	5 (10.9)	7 (13.7)	1 (12.5)	
	2	31 (24.2)	68 (30.0)	9 (19.6)	17 (33.3)	2 (25.0)	
	3	49 (38.3)	81 (35.7)	19 (41.3)	18 (35.3)	2 (25.0)	
	4	24 (18.8)	44 (19.4)	12 (26.1)	6 (11.8)	3 (37.5)	
	5	7 (5.5)	3 (1.3)	1 (2.2)	2 (3.9)	0 (0.0)	

**Table 2.** Mechanism of injury in predicting outcomes.

	Injury (*n* = 460)		Multicompartment injury (*n* = 460)		Injury out with clinical suspicion (*n* = 460)		30-Day mortality (*n* = 446)	
	OR	*P* value	OR	*P* value	OR	*P* value	OR	*P* value
FFS (*n* = 128)	1 (ref)		1 (ref)		1 (ref)		1 (ref)	
Fall > than standing (*n* = 227)	0.83 (0.52-1.31)	.437	1.53 (0.9-2.67)	.124	2.63 (1.18-6.69)	.026	0.72 (0.38-1.38)	.318
Unwitnessed fall (*n* = 46)	0.8 (0.4-1.65)	.544	1.11 (0.45-2.55)	.811	1.21 (0.25-4.55)	.793	2.31 (1.01-5.16)	.042
RTA (*n* = 51)	1.38 (0.67-2.94)	.39	2.71 (1.31-5.62)	.007	1.88 (0.53-6.18)	.302	0.57 (0.16-1.62)	.33
Other (*n* = 8)	0.79 (0.18-3.98)	.749	0.65 (0.03-3.92)	.696	0 (0 ->100	.987	<0.01 (NA->100)	.986

	**AOR**	***P* value**	**AOR**	***P* value**	**AOR**	***P* value**	**AOR**	***P* value**

FFS (*n* = 128)	1 (ref)		1 (ref)		1 (ref)		1 (ref)	
Fall > than standing (*n* = 227)	0.91 (0.56-1.46)	.690	1.65 (0.95-2.95)	.081	2.80 (1.23-7.28)	.021	0.70 (0.35-1.38)	.292
Unwitnessed fall (*n* = 46)	0.81 (0.40-1.67)	.561	1.13 (0.46-2.64)	.777	1.16 (0.24-4.41)	.837	2.66 (1.13-6.19)	.023
RTA (*n* = 51)	1.65 (0.78-3.64)	.203	2.63 (1.22-5.70)	.013	1.99 (0.54-6.89)	.277	0.71 (0.19-2.18)	.574
Other (*n* = 8)	1.14 (0.26-5.96)	.869	0.71 (0.04-4.51)	.756	<0.01 (<0.1->10)	.987	<0.01 (NA->10)	.986

## Results

### Demographics

In total, 465 patients were considered for this study. Five cases were excluded due to either not having IV contrast (1), or not covering the head, neck, chest, abdomen, and pelvis (4). The total number of patients included was 460.

In total, 128 participants underwent WBCT following FFS, 227 fall from greater than standing, and 46 a fall of unclear mechanism (unwitnessed group). Fifty-one participants suffered RTA and 8 had trauma CT for other indications. There was significant difference between these groups in terms of age, participants who suffered falls from standing having the greatest median age 85.5 years (Table 1). There were also disparities between the groups in terms of cognitive impairment, use of anticoagulation, and RTS.

### Compartments scanned

Table 1 contains a summary of the number of patients who had symptoms and/or signs in each radiological compartment as defined in the materials and methods section. For the whole cohort (*n* = 460), 1062/2300 (46.2%) radiological body compartments were scanned for which there was no clinical suspicion of injury (Table 1). In the FFS group (*n* = 128), 284/640 (44.4%) of radiological compartments scanned were done so in the absence of clinical suspicion of injury. The largest contributor to this was the pelvis, 73 pelvises were scanned in the absence of clinical suspicion of injury.

### Injuries

Table 2 demonstrates the analysis of mechanism of trauma compared with outcomes. A fall greater than standing was associated with a 180% increase in the risk of having an injury out with area of clinical concern (AOR 2.80, 95% CI 1.21-7.36; *P* = .021). There were significantly higher odds of multiple compartment injury in patients whose trauma was secondary to RTA compared with those who had an FFS (AOR 2.66, 95% CI 1.22-5.92; *P* = .013).

In the FFS group, 19.4% required active treatment for injuries found on WBCT, defined as any management measure excluding analgesia. There were 7 incidences of participants who had an FFS with injury out with the area of clinical concern (Table 3). None of these injuries required active treatment.

**Table 3.** Summary of injuries out with areas of clinical suspicion in the FFS group.

Suspected site(s) of injury)	Injury out with clinical suspicion	Compartment of injury out with clinical suspicion	Other injuries	Management of injury out with clinical suspicion	Days in hospital	RTS	Age
Head chest abdomen	Acute S3 sacral fracture	Pelvis	Thoracic spine fractures	Conservative	72	12	92
Neck chest	11 mm splenic laceration	Abdomen	Left haemopneumothorax and left rib fractures	Conservative	51	12	76
Head neck	Right 7th rib fracture	Chest	Facial fractures, humeral fracture	Conservative	55	12	90
Head neck chest abdomen	Left inferior and superior pubic ramus fracture	Pelvis	Left sided rib fractures	Conservative	6	12	85
Chest	Minimally displaced left anterior acetabular fracture	Pelvis	Left humeral head fracture	Conservative	12	12	96
Head	Pulmonary contusions and intra-articular humeral head fracture	Chest	Extra-axial haematoma and facial fractures	Conservative	39	8	71
Head	Moderate right pneumothorax and rib fracture	Chest	None	Conservative	30	12	91

### Outcomes

Predictors of mortality were assessed in Table 4 through multivariate analysis. Factors within the model with *P* value of <.1 (age, sex, and RTS) were included in the final model. Trauma score demonstrated a consistent increase in odds of 30-day mortality as the score fell. Patients with a trauma score of 8 had more than 1800% increased odds of 30-day mortality compared with those with a score of 12 (AOR 19.64, 95% CI 3-159.71; *P* = .0019). In this analysis, mechanism of injury was not demonstrated to be associated with 30-day mortality however as demonstrated in Table 2, patients who suffered unwitnessed falls had a higher mortality at 30 days compared with the FFS group (AOR 2.66, 95% CI 1.13-6.19; *P* = .023).

**Table 4.** Multivariate logistic regression of 30-day mortality in patients who underwent WBCT (*n* = 442).

		AOR	*P*
Age (per year)		1.05 (1.01-1.1)	.015
Sex	Female	1 (ref)	
	Male	1.86 (0.99-3.58)	.058
Revised trauma score	12	1 (ref)	
	11	6.67 (2.98-14.7)	<.001
	10	6.83 (2.13-20.62)	<.001
	9	15.12 (1.39-333.97)	.029
	8	19.64 (3-159.71)	.0012

There was no significant difference in mortality rate between all patients with injury on the WBCT (15.3%) and those without (10.6%) (Table 5). There was an increased risk of trauma-specific 30-day mortality.

**Table 5.** Comparison of outcome in participants with and without an injury detected on WBCT.

		Injury		
		No	Yes	
Days in hospital (*n* = 460)	Median (IQR)	2.0 (0.0 to 12.0)	11.0 (4.0 to 27.0)	<0.001
30-Day mortality (*n* = 446)	0	135 (89.4)	250 (84.7)	0.227
	1	16 (10.6)	45 (15.3)	
30-Day trauma mortality (*n* = 445)	0	150 (99.3)	262 (89.1)	<0.001
	1	1 (0.7)	32 (10.9)	

**Figure 2.**
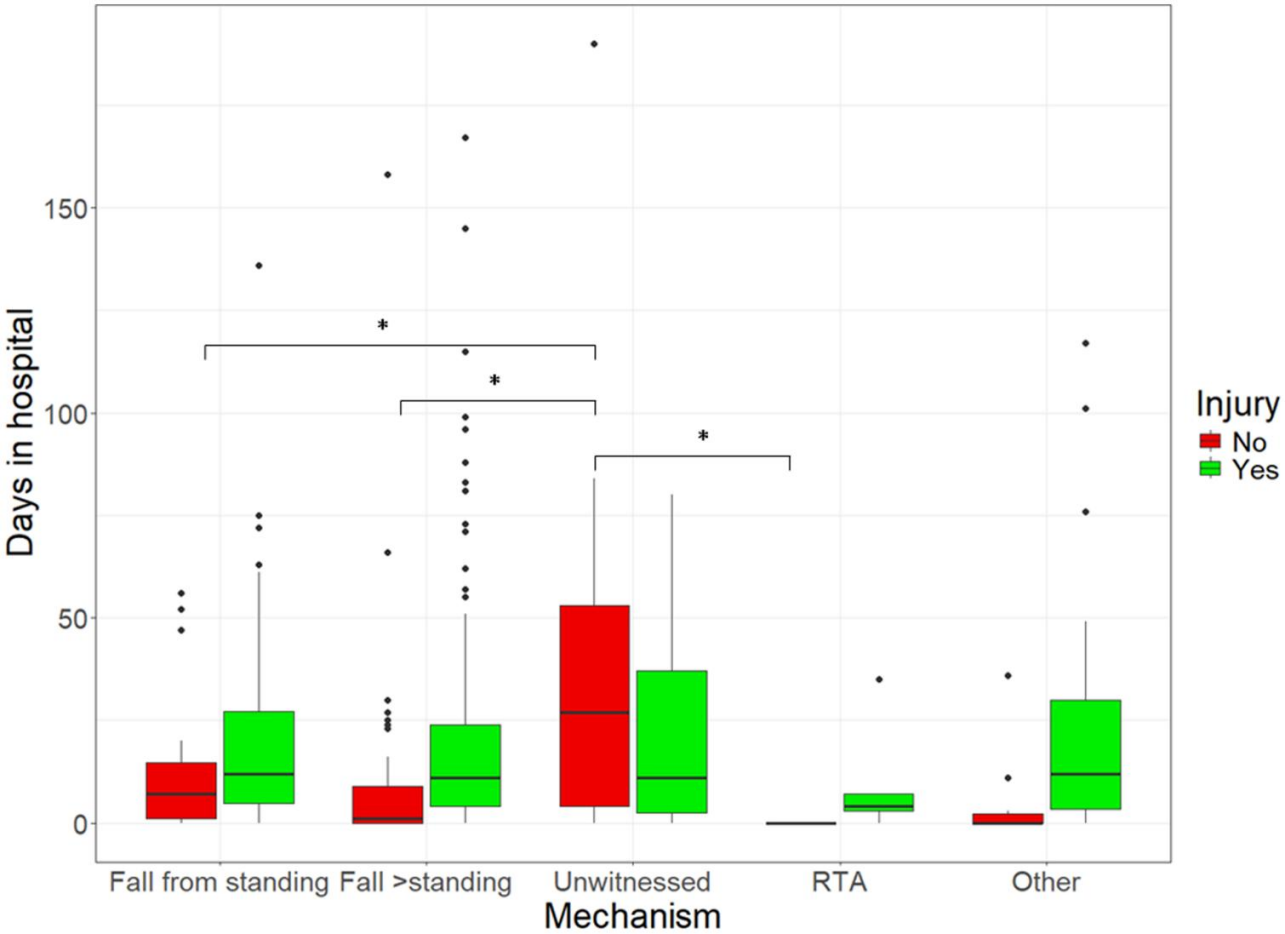
Comparison of days in hospital across injury groups. *P value <.05 on ANOVA. No injury unwitnessed vs No injury FFS adjusted *P* = .043. No injury unwitnessed vs No injury fall > standing adjusted *P* = .016. No injury unwitnessed vs No injury RTA adjusted *P* = .043.

Figure 2 assesses hospital stay following injury and demonstrates an increased stay for uninjured patients who had an unwitnessed fall compared with patients who had an FFS, fall from greater than standing, and RTA. There was no difference between hospital stay in patients who had an unwitnessed fall compared with other injury mechanisms when an injury was detected.

## Discussion

The primary objective of this study was to identify whether targeted CT imaging based on clinical suspicion in low impact trauma is a safe method in elderly trauma. Within our study, 7 of the 128 patients (5.5%) who fell from standing had an injury out with the area of clinical concern. All these injuries were managed conservatively. Two of the 7 injuries were more significant. One was an 11 mm splenic laceration and would likely have been detected on CT chest imaging. The other significant occult injury was a moderate sized pneumothorax which would have likely been detected on a chest X-ray (Table 3).

Patients who had a fall from greater than standing were significantly more likely to have an injury out with the area of clinical concern compared to the FFS cohort. These findings would support the notion that there is a low likelihood of injury out-with the area of clinical concern in the context of low impact trauma. There is limited data published on the correlation between clinical signs and symptoms and injuries in low impact trauma. In 2020, Hagan et al^13^ reported a negative predictive value of 99.7% for abdominal examination in predicting need for intervention following FFS, highlighting the importance of clinical history and examination in identifying injuries following low impact trauma.

The value of targeted scanning over WBCT within our institution could be measured in the number of CT compartments potentially saved. Based on the data in Table 1 in total 284 radiological compartments were scanned without clinical suspicion for those that fell from standing. The Royal college of Radiologists (RCR) currently recommends a consultant radiologist should report 12 examinations in a clinical session (4 h), meaning 23.67 consultant sessions could be saved over a 2-year period, or 11.84 sessions per year. This increases to 44.25 sessions per year if targeted scanning in all trauma patients >70 years old was performed. There would also be a benefit from reduction in radiation to patients,^14^ scanning time, and reduction in the environmental impact of imaging.

In 2016, the REACT 2 study reported no benefit to performing immediate WBCT in patients who suffered severe trauma compared with conventional imaging supplemented with targeted CT scanning.^15^ They found no reduction in hospital mortality in the WBCT group. Whilst we were unable to compare WBCT with targeted CT scanning in this study, our finding that the presence of injury did not influence 30-day mortality is in broad agreement with the data from the REACT 2 study (Table 5). Of the 151 patients who had a normal WBCT mortality at 30 days was 10.6%, compared with 15.3% in people who had any injury detected on WBCT (*P* = 0.227).

In 2021, Mohamed et al^16^ published a retrospective study comparing selective CT vs WBCT in elderly trauma patients. They reported a similar mortality at 30 days to the present study at 16.3% in their multiple injury group and 13.2% in the single injury group. Unlike the present study, they did not consider patients who did not have injury. As such the present study adds significantly to current evidence demonstrating that in elderly trauma, patients who do not have an injury detected on WBCT are still at high mortality risk.

Mohamed et al also reported that patients who had a WBCT at admission had a shorter hospital stay; however, it should be noted that in the WBCT group, there was a significantly higher number of patients who had suffered an RTA. We have demonstrated that mechanism of injury can influence length of hospital stay, and the observations of Mohamed et al should be interpreted with this in mind.

A large study published in surgery in 2013, by Dwyer et al,^9^ assessed over 13 000 patients who had a ground level fall and found that patients who underwent WBCT did not have reduced risk of mortality. This is of particular interest considering the present studies finding that injury on WBCT was not associated with increased 30-day mortality. In summary, at present, there is no decisive evidence that the utilization of WBCT in trauma reduces mortality.

Whilst Dwyer et al did not identify a reduction in mortality, they reported that WBCT reduced hospital stay. Whilst this is of clear importance to health care systems, it did not consider the burden to the imaging department. In particular, the time taken to report WBCT in low impact trauma delays reporting of potentially more pressing CT studies that are performed, thereby resulting in potentially significant reporting delays and backlogging of patients within the A&E department. Moreover, the present study demonstrated in the unwitnessed fall group no clear trend towards reduced hospital stays in those with no injury detected.^6^

The number of patients who have modifying treatments following WBCT can be modest with Salim and colleagues reporting a rate of 19% in 1000 patients with obvious evidence of injury following trauma.^17^ Our study found a similar rate of active treatment, with 19.4% of patients in the FFS group requiring active treatment. It should be noted however that in the present study, it was not a prerequisite that all patients should have external evidence of injury.

The weakness of this study relates to its design as a retrospective cohort study. There are likely to have been patients who had low impact trauma who did not meet the clinical threshold for WBCT and thereby would not have been captured by this study. Inclusion of such patients would have allowed comparisons between people who had targeted CT imaging and WBCT. This in turn would have afforded the ability to draw more clear conclusions regarding the value of WBCT following FFS with regards to reducing mortality.

There are several data points which may have been more easily collected in a prospective study design, specifically frailty scores and ISS scores. In terms of outcomes, we have been able to collect 30-day mortality, hospital stay, and injury; however, morbidity is also of significant importance which is challenging to assess retrospectively. To fully investigate the impact of WBCT on morbidity following trauma it would be of value to perform a randomised control trial which randomises elderly patients with an FFS to receive either WBCT in the emergency department or a more targeted clinical suspicion-guided CT. This study could fully analyse the impact of WBCT in low impact elderly trauma and would be of significant interest and importance.

The decision to include patients who had an unclear history of fall as a separate group, namely the unwitnessed fall group, may complicate the application of these results for clinicians. In some cases, patients may have had unwitnessed falls but were able to accurately recount the fall. Additionally, there are some patients who had a fall which was not witnessed but the position or place in which they were found strongly indicated a mechanism of fall. Within this study, we assessed whether a fall was unwitnessed based on the request for CT and the clinical notes at the time of admission to the emergency department. If the referrer regarding the mechanism of fall was unsure, we coded this as an unwitnessed fall. As such we would suggest that in the interpretation and application of these results, the method of identifying unwitnessed falls is kept in mind.

Finally, this study is of limited sample size, in particular the group of interest, namely people who suffered FFS, only 7 events of the primary outcome were captured (injury out with clinical suspicion). There is limited power therefore to detect differences in the primary outcome between this group and other groups such as those who suffered RTA, larger studies would help in elucidating these relationships.

In conclusion, falling from standing carries a lower risk of resulting in clinically occult injury in elderly patients. Targeted clinical suspicion-based approach to CT imaging in such patients should be considered. The current use of WBCT in elderly people who suffer FFS does not appear to detect clinically meaningful occult injuries, and therefore is not clearly justifiably with respect to the as low as reasonably possible (ALARA) principle set out by the ionising radiation medical exposure regulations (IRMER).

### Recommendations

1. Consider clinical suspicion-based targeted approach to imaging following fall from standing in elderly patients with a normal trauma score.
2. Low impact injuries in the elderly are associated with a high mortality even when trauma CT does not identify significant injuries.
3. In patients where the mechanism of injury is unclear, it should be noted that these patients are at particular high risk for mortality and may have factors other than trauma driving morbidity.
